# The Bitter Cry of Motherhood

**Published:** 1892-10-15

**Authors:** 


### The Hospital

An Institutional Journal of

Science, Medicine, Nursing, and Philanthropy.

*For Hospitals, Asylums, Infirmaries, Dispensaries, Medical Practitioners, Students, Nurses, and the Charitable Public.*

Vol. XIII.—No. 316.] [Oct. 15, 1892.

### The Bitter Cry of Motherhood.

Dr. Frederick H. Alderson, one of the vice-presidents of the West London Medico-Chirurgical Society, has published the story of a woman who, after bearing eleven children, died of her twelfth confinement because she had not obtained that skilled assistance which the least instructed of legally qualified medical men would have seen that she required, had he been present, and would have as promptly applied. Such cases are quite every day occurrences : to the true humanitarian they are painful and distressing to the last degree. Let every adult reader pause for a few moments and consider what child-bearing really is. It is the most strenuous of all the agonies which human nature is called upon to endure. No one who thinks can fail to be struck by the fact that the first historians of the human race were profoundly impressed by the painful travail of childbirth. "In sorrow shalt thou bring forth children," was the doom which a religious chronicler believed to have been pronounced upon Eve. Man knows no agony that can be compared with that which woman endures in giving birth to her children. This circumstance, though physiological and natural, is, as all medical men know, attended by numerous possible dangers of the most fatal kind. Women themselves, who have borne many children, are unceasingly haunted by the presence in their minds of these dangers. To such women the period of every additional pregnancy is like "the valley of the shadow of death." Indeed, if we will but consider it, the coming forth of every mother from her chamber of travail is like the miracle of the resurrection of the dead.

It is well that medical men should sometimes compare certain parts of their work with certain other parts, and should have in their minds some clear standard of the relative importance of the different parts. As a matter of fact, all men in family practice do thus compare; and it is very significant that every family practitioner very soon learns to regard his midwifery cases as the most important part of his practice. For these he stays at home week after week when his own health is breaking down for want of his much-needed holiday; for these he permits himself to be deprived of his nightly sleep, and makes no complaint; if these go well he is prosperous and cheerful; if they go ill he sees the spectre of ruin in the near distance. There can be no doubt whatever of the relatively important part which midwifery plays in the practice of the average medical man.

It is the family practitioner who really does the medical work of the world; and that which is relatively the most important part of his practice is, in truth, the most important of all medical practice. If anyone doubts this he has but to consider the mere extent of the work. The average practitioner may have one case of consumption every twelvemonth, but he will have a hundred cases of midwifery : and let it never be forgotten that every case of midwifery, in its possibilities, is truly and essentially a "dangerous case." It is not affirmed that childbirth is pathological and abnormal; it is admitted that it is physiological and normal ; nor is it denied that nature has made wonderfully varied and complete provision for ordinary labour. Nevertheless, even ordinary childbirth is in itself so extraordinary a thing that, though it be natural, it is to the reasoning and philosophic mind a real and true "convulsion of nature." Many a time and oft has the present writer said to himself and to others that if the fact of childbirth had never been known in experience no man of science in the world could have been persuaded to believe in its possibility.

What is wanted at the present moment is that the voting and law-making part of the world shall understand the facts and circumstances of childbirth as doctors understand them. And the reason why these facts and circumstances should be so intelligently understood is that all women, suffering women, at these tremendous crises in their lives, ought to have all the help and means of safety which an enlightened science and a cultured humanitarianism can possibly give to them. We are all coming to recognise now—are we not ?—that women who play the part of wives and mothers in the world are in a certain measure not only fulfilling high destinies, but deserving the best that is possible at the hands both of God and of men. Doctors see this very clearly, and those doctors whom Nature and science have made chivalrous cannot be content unless women as a class at the time of childbearing are provided with every means of safety and restoration to health which science and humanitarianism can devise.

The voters and law-makers of our country must try to understand this question. They will be called upon to deal not only with it, but with a good many other half-scientific and half-humanitarian questions in the near future. They must begin by divesting themselves of their prejudices, and by trying to use the methods of science. Those methods in their essence consist of nothing more, and nothing less, than the getting at the true facts of the case, and judging rightly among them. Now, there is only one way of getting at the true facts of this particular case of skilled help for women in childbirth, and that is by going to the fountain head—the trustworthy and trusted members of the medical profession.

Dr. Alderson in his pamphlet, based on the sad case we started with, contends for the State regulation and control of midwives. His argument, if we understand it aright, is—that midwives should either be properly trained, or altogether disallowed. The argument seems to us to be as sound and as obvious as the contention that two and two make four. Nothing can be more obvious than this, that wherever there is important work to be done the only rational policy is to employ thoroughly competent persons to do it. We have made this very broad and strong assertion, that in all the world there is no circumstance more profoundly significant than the circumstance of childbirth; and in all the world there is no work more really important in itself than that of skilled ministration to child-bearing women. Science, at least the broad and liberal science of modern times, joins with the most cultured humanitarianism in reverencing this profound mystery, and in offering to those women who are chosen by Providence to be its depositaries, all the tender sympathy and all the most enlightened and instructed help which the generous times can command.

The immediately practical question of the moment is this: Should the present race of midwives be recognised and registered? That is a question which seems to be somewhat premature. That some of them might properly be registered few will care to deny; that many of them are altogether unfit to be entrusted with the responsibilities which registration would confer upon them no well-informed person would dare to affirm. But that the question of the proper training of those who take charge of women in childbirth is ripe, not only for discussion but for decision, is obvious to us all. It is very far from creditable to our science that this most extensive field of practical scientific work should be left so largely in the hands of persons who are not only unskilled, but worse than unskilled; who have knowledge enough to make them rashly confident, but not knowledge enough to make them conscious of their own ignorance. As little is it to the credit of our religion and our philanthrophy. If we could but see in a mass all the misery and all the loss of life which result from this state of things, even in a single year, we should be filled with amazement and horror. It is true that the medical profession has secured the rejection of certain proposed remedial measures in the past; but it is not true that the medical profession is opposed to all legislation on the subject. The opposite is rather the truth. The worthiest medical men recognise it to be their duty to inform the public mind upon the question, and to press for remedial measures which shall be at once inteligent and effectual. It seems to us to be the immediate duty of the opposed sections of the medical profession to unite, and to press upon the Legislature, if not the registration of the fit, which is comparatively a minor matter, at least the disqualification, or better training, of the unfit.

